# Mesenchymal stem cell-derived extracellular vesicles in skin wound healing: the risk of senescent drift induction in secretome-based therapeutics

**DOI:** 10.1186/s40779-023-00498-0

**Published:** 2023-11-30

**Authors:** Anna Smirnova, Elena Yatsenko, Denis Baranovskii, Ilya Klabukov

**Affiliations:** 1 National Medical Research Radiological Center, Obninsk, 249036 Russia; 2 Obninsk Institute for Nuclear Power Engineering, National Research Nuclear University MEPhI, Obninsk, 249031 Russia

**Keywords:** Cell therapy, Inflammaging, Secretome, Senescence

Regulatory changes in senescent cells could potentially affect the composition of extracellular vehicles (EVs), specifically altering their size and cargo. As a result, the released senescent EVs contain an unpredictable cocktail of growth factors and cytokines. These biomolecules have dual effects, potentially guiding the induction of senescence in affected cells and promoting an inflammation-related “domino effect” within the cellular environment, ultimately leading to tissue inflammaging.

Based on this view, we read with great interest the paper by Ding et al. [1] regarding the key biomedical issues of applying mesenchymal stem cell (MSC)-derived EVs for skin wound treatment. EVs secreted by cells have gained significant attention in recent years due to their potential therapeutic applications. These microvesicles are believed to play a crucial role in intercellular communication and have been investigated for their ability to deliver therapeutic cargo to target cells. However, we suppose it is important to critically evaluate in more detail the potential risks associated with EVs applications that may induce the senescence drift in recipient’s cells.

The authors noted the heterogeneity of EVs including size, yield and quantity, contents, and functional effects on recipient cells. Indeed, variations in isolation techniques, purification protocols, and storage conditions can significantly affect the quality and potency of EVs. The lack of standardization raises concerns about the reproducibility and reliability of EV-based therapies, making it difficult to compare results from studies with different designs.

MSCs derived from different sources, such as bone marrow, adipose tissue, and umbilical cord, display distinct differentiation tendencies, secrete unique paracrine factors, and have different immunomodulatory capabilities [2]. A recent study has shown that MSCs may be characterized by a senescence phenotype and reinforced growth arrest, termed the “senescence-associated secretory phenotype” [3]. The secretome of MSCs with senescent phenotypes generates a proinflammatory microenvironment affecting surrounding cells [4]. Human MSCs could enter the senescence phenotype, during expansion passing or cryopreservation [2]. Injections of the proinflammatory secretome of senescent cells could potentially induce a sequence of processes known as “inflammaging” in the affected tissues, consequently resulting in side effects.

Although scientific and clinical efforts have failed to develop a universal diagnostic kit for senescent cells, there are currently various laboratory assays available for the evaluation of the senescence components of EVs in clinical practice. These assays include enzyme-linked immunosorbent assay, which enables accurate assessment of a wide range of proteins, and matrix-assisted laser desorption/ionization mass spectrometry, which provides an approximate assessment through shotgun analysis. It is worth noting that there is a lack of affordable and precise methods to quantify the composition of cellular secretomes. However, advanced microfluidic-based cell assays may provide a potential solution.

Therefore, inflammaging associated with the secretome may pose a challenge for the clinical applications of EVs derived from the secretome of multipotent cells [5]. Prior to their use in therapy, it is crucial to assess the immunomodulatory potential of MSCs and senescent cell content, determine the functionality of isolated MSCs [2], and analyze the composition of MSC-derived secreted proteins.

The perspective we offer is expected to shed light on the impact of cell secretion and prevent potential complications and adverse events associated with the cellular senescent phenotype in EV-based therapies.

## Data Availability

Not applicable.

## Abbreviations

EVs: Extracellular vesicles
MSC: Mesenchymal stem cell
